# Identification of a DNA region associated with the cool virulence of *Ralstonia solancearum* strain UW551 and its utilization for specific detection of the bacterium’s race 3 biovar 2 strains

**DOI:** 10.1371/journal.pone.0207280

**Published:** 2018-11-14

**Authors:** Michael J. Stulberg, Xueqing Cai, Abdelmonim Ali Ahmad, Qi Huang

**Affiliations:** 1 Floral and Nursery Plants Research Unit, U. S. National Arboretum, U. S. Dept. of Agriculture-Agricultural Research Service, Beltsville, MD, United States of America; 2 Department of Plant Pathology, Faculty of Agriculture, Minia University, El-minia, Egypt; Dong-A University, REPUBLIC OF KOREA

## Abstract

The cool virulent *Ralstonia solanacearum* race 3 biovar 2 (r3b2) strains cause destructive brown rot of potato. They are quarantined pathogens in Europe and Canada and select agent pathogens in the United States. We previously identified r3b2 (sequevars 1 and 2)-unique fragments that clustered into 32 regions in the genome of *R*. *solanacearum*. In this study, we targeted five of those regions for mutagenesis in order to determine whether they are involved in cool temperature-related biological functions for diagnostic purpose. Knockout mutants of four regions produced no changes to the biology of the r3b2 strain UW551. The mutation of region 13, which is 3,407 bp in size, resulted in significantly reduced twitching motility, attachment to the roots of tomato seedlings, and virulence under cool temperature conditions (18–24°C), although no significant difference was found under warm temperature conditions (24–30°C) as compared to the wild type strain. As a result, we designed primer pair Rs-CV-F and Rs-CV-R to target the region 13 for specific detection of r3b2 strains of *R*. *solanacearum*. Our assay specifically detected all the 34 r3b2 strains and none of the 56 non-r3b2 strains of *R*. *solanacearum*, nor any other five plant- or soil-associated bacteria including *Enterobacter cloacae*, *Pseudomonas syringae* pv. *syringae*, *Xanthomonas campestris* pv. *campestris*, *X*. *citri*, and *R*. *pickettii*. Unexpectedly, in silico analysis predicted that a recently deposited non-sequevar 1 or 2 Brazilian *R*. *solanacearum* strain RS489 would be recognized by our assay and by previously published r3b2-specific assays, although the cool-virulent status of this strain is unclear. Our PCR assay is the first to target a DNA region associated with cool-virulence that makes r3b2 strains highly regulated pathogens for specific detection of this important group of *R*. *solanacearum*.

## Introduction

*Ralstonia solanacearum* causes devastating plant diseases common in tropical, subtropical and temperate regions of the world. One phylogenetically coherent and largely clonal group of *R*. *solanacearum*, known as race 3 biovar 2 (r3b2) and belonging to phylotype IIB sequevars 1 and 2 (IIB-1&2), causes highly destructive brown rot of potato. Unlike other tropical strains of *R*. *solanacearum*, r3b2 strains are well adapted to temperate climates and are quarantined pathogens in Europe and Canada. They are also listed as select agent pathogens in the United States [1] because they are capable of surviving and infecting potatoes in seed potato producing areas, thereby potentially threatening U.S. agriculture. Although the terms race and biovar are used to define this group of *R*. *solanacearum*, these terms are only correlative to the cool virulent nature of the r3b2 strains. Little is known about the underlying molecular basis for the cold tolerance and cool virulence of the r3b2 strains in potato that makes them highly regulated. As a result, it has been a challenge for over two decades to identify and target genetic regions linked specifically to cool virulence of r3b2 strains for specific detection of this important group of *R*. *solanacearum*.

There have been a few recent studies comparing cool virulent and non-cool virulent *R*. *solanacearum* strains during the infection process at different temperatures in the hopes of elucidating specific cool-virulence factors. Bocsanczy et al. [2] found that r3b2 IIB-1&2 strains are not uniquely cool virulent, since some race 1 biovar 1 (r1b1) phylotype IIB sequevar 4 strains in Florida can also infect and wilt potato and tomato plants at 18°C, although the r1b1 strains were not as virulent in potato as the r3b2 IIB-1 strains at this temperature. They also found that twitching motility in non-cool virulent strains was attenuated at 18°C but unaffected in the cool virulent strains, suggesting that maintaining twitching is important to cool virulence of *R*. *solanacearum*. Their subsequent proteomic comparison study of *R*. *solanacearum* strains revealed putative involvement of temperature dependent virulence factors including twitching motility porin (PilQ) and two components of a putative type VI secretion system [3]. A transcriptome and mutational analysis by Meng et al. [4] identified a small set of genes, including a mannose-fucose binding lectin and two quorum sensing-regulated genes of unknown functions, differentially expressed by temperature, contributed significantly to the onset of disease from r3b2 strains at cool temperatures. It is still largely unknown, however, what other genes and/or regulatory elements are responsible for the cool virulence of the r3b2 IIB-1&2 strains in potato. Such information is urgently needed in order to accurately define the r3b2 strains of *R*. *solanacearum*, to use the cool virulence genes/elements as diagnostic markers for reliable detection and exclusion methods, and to improve our understanding of the cool virulent nature of the r3b2 IIB-1&2 strains for the development of effective strategies to control this important group of *R*. *solanacearum*.

Genome sequencing enables comparative genomics between closely related strains. We recently did comparative genomics to identify unique DNA sequences in r3b2 IIB-1&2 strains compared to the other sequevars of *R*. *solanacearum* [5]. Amajority of the unique fragments identified are adjacent to or code for hypothetical proteins with no known function. We identified 32 regions of variable sizes of the *R*. *solanacearum* genome (chromosome and megaplasmid) that contain long-continuous or short-interspersed unique sequences, and we targeted five regions for knockout by double homologous recombination. The knockouts were compared with their wild type (wt) under both cool and warm temperature conditions in *in vitro* growth, motility, root attachment, and virulence. We also designed and validated PCR primers targeting a r3b2 cool virulence-related region for specific detection of the r3b2 strains of *R*. *solanacearum*.

## Materials and methods

### Bacterial strains and plasmids

Bacterial strains and plasmids used and constructed in this study are listed in Tables 1 and 2.

**Table 1:** Bacterial strains and plasmids used in this study.

Designation	Relevant characteristics^a^	Source
**Strains**		
*Ralstonia solanacearum*		
UW551	Wild-type, race 3 biovar 2, phylotype IIB sequevar 1	C. Allen, USA
UW551ΔR13	UW551 Region 13::*aacC1* Gm^R^	This study
UW551ΔR27	UW551 Region 27::*aacC1* Gm^R^	This study
UW551ΔR9	UW551 Region 9::*aacC1* Gm^R^	This study
UW551ΔR15	UW551 Region 15::*aacC1* Gm^R^	This study
UW551ΔR23	UW551 Region 23::*aacC1* Gm^R^	This study
*Escherichia coli*		
TOP10	F- *mcr*A Δ(*mrr*-*hsd*RMS-*mcr*BC) Φ80*lac*ZΔM15 Δ*lac*Χ74 *rec*A1 *ara*D139 Δ(*ara*-*leu*)7697 *gal*U *gal*K *rps*L (StrR) *end*A1 *nup*G	Invitrogen
TP997	MG1655 *lacIP*Δ::*bla-aadA1148 galK*::*aacC1067*	Addgene
**Plasmid**		
pCR Blunt II TOPO	PCR cloning vector, Kan^R^ Zc^R^	Invitrogen
pCR Blunt II-Gm	pCR Blunt II TOPO with a 616-bp Gm cassette, Gm^R^ Kan^R^ Zc^R^	This study
pCR Blunt II-Up-Gm- Down	pCR Blunt II-Gm with approximately 1-kb fragment upstream and 1-kb downstream of each of the targeted *R*. *solanacearum* regions inserted before and after the Gm cassette, Gm^R^ Kan^R^ Zc^R^	This study

^a^Gm^R^, Kan^R^, and Zc^R^ indicate resistance to gentamicin, kanamycin, and zeocin, respectively.

**Table 2:** Bacterial strains used in this study for testing of PCR specificity using primer set Rs-CV-F/Rs-CV-R.

Strain (Alt ID)	Biovar; Phylotype/ Sequevar	Rs-CV-F/ Rs-CV-R	Strain (Alt ID)		Biovar; Phylotype/ Sequevar	Rs-CV-F/ Rs-CV-R
*Ralstonia solanacearum species complex strains* *Select agents (r3b2 phylotype IIB seq 1 and 2)*			*Non-select agents continued*			
UW120 (S214)	2; II	✓	P506		1; IIB/4	✕
UW150 (S243, Hayward0137a)	2; II	✓	P673		1; IIB/4	✕
UW220a	2; II	✓	P618 (99.1120/1)		1; IIB/4	✕
UW224 (Harris 220)	2; II	✓	P597		1; IIA/37	✕
UW257 (Gonzalez G-13)	2; II	✓	P550		1; IIA/7	✕
UW260	2; II	✓	RUN060 (JT525)		1; III/19	✕
UW276	2; II	✓	RUN074 (Molk2, R633)		1; IIB/3	✕
UW344 (10 1SC)	2; II	✓	UW119 (S213)		3; I	✕
UW425 (O249)	2; II	✓	Rs121		3; I	✕
UW449 (CIP259)	2; II	✓	Pss32		3; I	✕
UW492 (CIP302)	2; II	✓	Pss530		3; I	✕
UW501 (CIP181)	2; II	✓	Pss4		3; I/15	✕
UW551 (I-35)	2; IIB/1	✓	Pss266		3; I	✕
UW552	2; II	✓	Pss97		3; I	✕
Pss1370	2; II	✓	Pss185		3; I	✕
Pss1475	2; II	✓	Pss201		3; I	✕
Pss1586	2; II	✓	Pss278		3; I	✕
RUN160 (JT516)	2; IIB/1	✓	Pss221		3; I	✕
RUN147 (CMR34, CFBP7029)	2; IIB/1	✓	Pss106		3; I	✕
RUN256 (PSS525)	2; IIB/1	✓	Pss73		3; I	✕
RUN141 (CMR24, CFBP7027)	2; IIB/1	✓	Pss18		3; I	✕
RUN440 (RE)	2; IIB/1	✓	Pss71		3; I	✕
RUN035 (IPO1609)	2; IIB/1	✓	HB512		3; I	✕
IVIA1602.1	2; II	✓	JS526		3; I	✕
4155	2; II	✓	GZ519		3; I	✕
4153	2; II	✓	FJ47		3; I	✕
NCPPB2505	2; II	✓	GX53		3; I	✕
NCPPB1584	2; II	✓	JS526		3; I	✕
CSL Pr 3467 (P.6019)	2; II	✓	RUN054 (GMI1000, JS753)		3; I/18	✕
CSL Pr 3468 (P.6018)	2; II	✓	UW151 (S244, Hayward 092)		4; I	✕
CSL Pr 1328	2; II	✓	Pss191		4; I	✕
UW80 (S206, CIP309)	2; IIB/2	✓	Pss565		4; I	✕
RUN628 (CFBP3879, CFBP1414)	2T; IIB/2	✓	Pss901		4; I	✕
RUN461 (CFBP1410, K164)	2T; IIB/2	✓	Pss51		4; I	✕
*Non-select agents*			Pss228		4; I	✕
UW349 (23-10BR)	2T; IIB/27	✕	Pss114		4; I	✕
RUN083 (PSI07, CFBP7288)	2T; IV/10	✕	Pss267		4; I	✕
RUN133 (CMR15, CFBP6941)	2T; III/29	✕	Pss262		4; I	✕
UW9 (S147)	1; IIB/3	✕	Pss1655		4; I	✕
UW25 (K60)	1; IIA/7	✕	Pss1283		4; I	✕
Rs5	1; IIA/7	✕	RUN062 (BDB)		IV/10	✕
Rs116	1; II	✕	RUN088 (*R*. *syzygii*)		IV/9	✕
Rs124	1; II	✕				
Rs126	1; II	✕	*Non-R*. *solanacearum species complex strains*			
Rs129	1; II	✕	*R*. *pickettii*			✕
RUN302 (IBSBF1503)	1; IIB/4	✕	*Enterobacter cloacae*			✕
RUN651 (LNPV24.25)	1; IIB/4	✕	*Xanthomonas campestris* pv. *campestris*			✕
P446	1; IIB/4	✕	*Pseudomonas syringae* pv. *syringae*			✕
P487	1; IIB/4	✕	*X*. *citri*			✕

### Growth of bacterial strains and DNA extraction

To grow *R*. *solanacearum*, the bacterium was freshly streaked from a frozen stock, and a single colony was picked and grown as described before [5]. *R*. *solanacearum* inocula for biovar testing, motility assays, and plant inoculation were also prepared, and final inoculum cell concentration for plant inoculations confirmed as described before [5]. *Escherichia coli* strains were cultured at 37°C in Luria-Bertani medium [6]. When needed, antibiotics were added at 50 μg/ml for ampicillin, 25 μg/ml for kanamycin, and 15 μg/ml for gentamicin. Total DNA was extracted using Qiagen’s Blood and Tissue Kit (Qiagen, Chatsworth, CA) following the manufacturer’s instructions.

### Grouping and mapping of r3b2-unique sequences into clustered regions in *R*. *solanacearum* strain UW551

Unique fragments identified previously through comparative genomics [5] were mapped onto the *R*. *solanacearum* strain UW551 genome using the free online A plasmid Editor (ApE) and Artemis. These programs helped visualize 32 regions that contain all of the 115 500-bp unique sequences. Five regions with various sizes were targeted for knockout studies, using ApE to identify primers in flanking genomic sequence for cloning purposes.

### Primer design and PCR conditions

Primers used to generate the approximately 1 kb fragments upstream and downstream of each of the targeted regions for cloning were designed based on the deposited UW551 draft genome sequence in GenBank (AAKL00000000.1). The selected regions were entered into the ApE program for primer design and mapping graphics. Similar design parameters (GC = 45 to 60%, Tm = 60 to 64°C, primer length 18 to 26) were used, and different amplicon sizes were chosen for the intended purpose (i.e. specific detection, generation and confirmation of mutants). The specificity of each primer pair and amplicon was checked by BLASTn against the UW551 genome for mutagenesis, and against the nr and WGS databases in GenBank for specific detection of r3b2 (IIB 1&2) strains of *R*. *solanacearum*.

For mutagenesis, PCR was performed in a 20 μL volume containing 1 x HiFi HotStart ReadyMix (Kapa Biosystems, Boston, MA), 5 pmol of each primer (S1 Table), and 20 ng of bacterial genomic DNA with the following thermocycling parameters: 95°C for 3 minutes, 30 cycles of 98°C for 20 seconds, 58°C for 15 seconds, and 72°C for 30 seconds, with a final 2-minute extension at 72°C.

For detection, the primer pair Rs-CV-F, 5’-CATAGCGGTCTCAGCAGGGTC-3’, and Rs-CV-R, 5’-GTTCTGCCGGTTAGACGACGTG-3’ target a r3b2-unique sequence in region 13 of *R*. *solanacearum* UW551 and was tested against the same collection of 95 bacterial strains used previously [5]. The collection includes 34 r3b2 and 56 non-r3b2 strains of *R*. *solanacearum*, as well as 5 other plant- or soil-associated bacteria, including *E*. *cloacae*, *P*. *syringae* pv. *syringae*, *X*. *campestris* pv. *campestris*, *X*. *citri*, and *R*. *pickettii* (Table 2). The PCR was performed in a 20 μL volume containing 1 x GoTaq Green master mix (Promega) with 10 pmol of each primer and 30 ng of bacterial genomic DNA with the following thermocycling parameters: 94°C for 4 minutes, 35 cycles of 94°C for 1 minute, 60°C for 1 minute, and 72°C for 1 minute, with a final 10-minute extension at 72°C.

### Construction of *R*. *solanacearum* UW551 mutant strains

Targeted regions (Table 3) containing r3b2-unique sequences in the wt strain UW551 of *R*. *solanacearum* were deleted by homologous recombination using the suicide vector pCR Blunt II TOPO (Invitrogen). A 616-bp gentamicin cassette was first amplified from a colony of TP997 (Addgene, Cambridge, MA), by PCR with primers 5′-CGAATCCATGTGGGAGTTTA-3′ and 5′-TTAGGTGGCGGTACTTGGGT-3′ [7]. The cassette was cloned into the TOPO site of the vector pCR Blunt II TOPO using Invitrogen’s Zero Blunt TOPO PCR Cloning Kit according to the manufacturer’s instructions to generate pCR Blunt II-Gm. Two regions of DNA approximately 1 kb in size, located upstream and downstream of the target region, were then amplified by PCR using primers in S1 Table, digested with respective restriction enzymes, and cloned sequentially into the multiple cloning sites before and after the gentamicin cassette in pCR Blunt II-Gm to obtain pCR Blunt II-Up-Gm-Down. The resulting plasmid was electroporated into competent cells of *R*. *solanacearum* strain UW551 using a MicroPulser Electroporator (Bio-Rad) in 0.1 cm pulsar cuvettes with settings of 1.0K V and 4 ms. This was followed by selection on gentamicin-containing triphenyltetrazolium chloride (TZC) plates [8] for strains that had undergone homologous recombination between Up-Gm-Down on pCR Blunt II-Up-Gm-Down and genomic DNA. Electrocompetent cells of *R*. *solanacearum* were prepared as described previously [9].

**Table 3:** Characterization of mutants of *R*. *solanacearum* UW551 generated by replacing the targeted regions with a gentamicin cassette through homologous double recombination.

Name of mutant	Biovar	Targeted region	Location of the targeted region in UW551 contig or UY031 genome	Size (bp) of the targeted region
UW551ΔR13	2	Region 13	Contig_0581 (70988–74394)	3,407
UW551ΔR27	2	Region 27	Contig_0581 (133556–135158)	1,603
UW551ΔR9	2	Region 9	UY031 (2289712–2308305)	18,594
UW551ΔR15	2	Region 15	Contig_0535 (1229–7370)	6142
UW551ΔR23	2	Region 23	Contig_0571 (80694–81871)	1,178

### Biovar test

To determine whether the deletion of targeted regions in *R*. *solanacearum* resulted in any changes in the mutants’ biochemical properties, biovars of the mutant strains were determined using the improved biovar test [10].

### Plant growth and inoculation

Seeds of tomato (*Lycopersicom esculentum* Mill. cv. “Bonnie Best”) were planted in Sun Gro Metro-Mix 360 growing medium (Sun Gro Horticulture, Agawam, MA) and grown on a mist bench in a greenhouse section at 22°C with 14 h of light until germinated. They were then moved to a secured greenhouse section or growth chambers approved for select agent research, transplanted into 12.5-cm pots at four to six true leaves, and inoculated two to seven days later. Inoculations were performed by soil drenching; 40 ml suspensions of 2 x 10^7^ to 1 x 10^8^ cells of *R*. *solanacearum* were poured into each pot. Plants were kept at two different temperatures: 'warm', between 24°C (night) and 30°C (day) and 'cool', between 18°C (night) and 24°C (day) with 14 h of light daily. Disease symptoms were recorded twice a week for four weeks, after which, asymptomatic plants were processed using the method of Stulberg and Huang [11] to determine whether the plants were latently infected by *R*. *solanacearum*.

### In vitro growth of *R*. *solanacearum* strains

To compare in vitro growth of the wt and mutant strains of *R*. *solanacearum* at different temperatures, the overnight culture of each strain was diluted to an OD_600_ of 0.01 in 30 ml of casamino acid peptone glucose broth [12], and grown for 24 h at 28°C and 64 h at 21°C with shaking. At indicated time intervals, one 500 μl aliquot was removed, from which three 100 μl volumes were measured for their OD_600_ values in Tecan’s Infinite F50 plate reader (Tecan Group Ltd., Switzerland) for each strain. The experiment was repeated three times.

### Motility assays

The swimming, swarming, and twitching motilities of *R*. *solanacearum* strains at warm (28°C) and cool (21°C) conditions were examined using previously described methods [13]. Each assay was repeated three times with three replicates per strain per assay.

### Root attachment assay

The assay was performed based on the method of Dalsing and Allen [14] with modifications. Briefly, tomato seeds were surface sterilized with 10% bleach for 5 min, placed on 1% agar plate and grown for 4 days at 28°C. Ten microliters (2 x 10^8^ CFU/ml) of the wt strain UW551 or the mutant strain UW551ΔR13 of *R*. *solanacearum* were placed on 3 cm sections of individual seedling roots to deliver 6.6 x 10^5^ CFU/cm of root. After incubation for 2 h, inoculated root segments were excised, gently mixed for 10 s in 15 ml conical tubes of sterile water, and blotted dry. Batches of ten 3 cm root segments were pooled, ground, serially diluted, and the 1:100 and 1:1000 diluted extracts were plated on TZC plates to quantify CFU of each strain attached per cm of root. The root attachment assay was performed under two temperature conditions: 28°C (warm) and 20°C (cool). The assay under each temperature was performed three times.

### RNA isolation and gene expression analysis

Total RNA was isolated from three milliliter-overnight culture of *R*. *solanacearum* wt UW551 and mutant UW551ΔR13 grown at 20°C and 28°C using Qiagen’s RNeasy Protect Bacterial and RNeasy Mini Kits (Qiagen, Inc., CA) according to manufacturer’s protocol. Contaminating DNA was removed from isolated RNA and one microgram of total RNA was reverse transcribed as described before [15]. Quantitative PCR was carried out as described by Ahmad, Stulberg & Huang [15] with gene-specific primers normalized to 16S rRNA using primers designed previously for 16S rRNA [16], *pilQ* [2], and *pilT* [15]. Relative gene expression levels were determined as described before [15] and the experiment was performed two times with three technical replicates each.

### Statistical analysis

Virulence was estimated by calculating a colonization index (CI) based on the number of wilted and latently infected plants [17] as described before [5]. CI was calculated from an initial trial of plants in cool and warm conditions. If any difference was noted, additional trials were conducted at both temperatures. For two mutants, UW551Δ9 and UW551Δ27, additional trials were done as controls for mutant strains. CI was calculated from the total plants tested per mutant and compared to controls run only during those same trials. Virulence data were analyzed by one-way ANOVA using web-based statistical software (http://vassarstats.net/anova1u.html). Means were compared using the Tukey's Honest Significant Difference test provided by the software. The means of growth rate, swimming, swarming, and root attachment assays between the UW551 wt and mutant strain UW551ΔR13 of *R*. *solanacearum* were analyzed for significant differences using the t test in Microsoft Excel.

### Sequevar analysis

The sequevar status of *R*. *solanacearum* strains RS 488 and RS 489 [18] was determined based on their endoglucanase sequences using the *R*. *solanacearum* typing computer program [19] or by constructing a phylogenetic tree [5,20].

## Results

**Fig 1:**
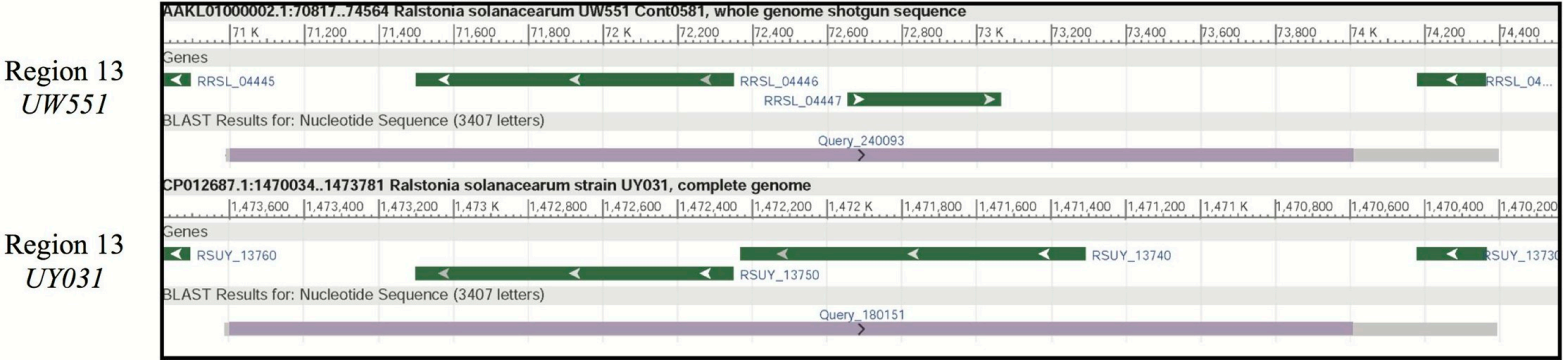
BLASTn results of region 13 against two r3b2 genomes: UW551 and UY031. The title of each result contains the name of the strain, contig and nucleotide position of the region depicted, and genes are shown in green with the gene IDs. A thick gray bar at the bottom of each BLASTn result represents a match to the queried sequence. The purple bar highlights the r3b2 unique sequence in these regions.

We previously identified DNA fragments unique to the r3b2 (IIB-1&2) strains of *R*. *solanacearum* [5]. When mapped to the genome sequences of the r3b2 strains UW551 and UY031, these fragments grouped into 32 genomic regions. These regions contain either single or multiple r3b2-unique DNA fragments (purple bars along query, Fig 1), adjacent to or containing mostly genes with unknown function. Five regions were targeted for deletion (Table 3), of various size and number of genes in the regions, to determine if any are associated with the cool virulence of *R*. *solanacearum* strain UW551. All five mutants of *R*. *solanacearum* retained a biovar 2 identity (Table 3), suggesting that none of the regions impact the strain’s ability to utilize a carbohydrate panel [10].

The five mutants were tested for virulence under both cool and warm temperature conditions (Table 4). Under cool conditions, virulence measured by colonization index (CI) was significantly lower for the mutant strain UW551ΔR13 (0.40) compared to wt r3b2 strain UW551 (0.80), yet not significantly different from the tropical control strain K60 (0.22) (Table 4). Under warm conditions, however, the CIs of UW551ΔR13, wt r3b2, and non-r3b2 strains were not significantly different (0.72, 0.78 and 0.56, respectively) (Table 4). None of the other mutants displayed a significant change in CI from the wt r3b2 control at both cool and warm temperatures (Table 4).

**Table 4:** Virulence of *R*. *solanacearum* strains in tomato plants at warm and cool temperature conditions.

Strain	Colonization Index (CI)	
	Cool (18–24°C)	Warm (24–30°C)
UW551	0.80^a^	0.78^a^
K60	0.22^b^	0.56^a^
UW551ΔR13	0.40^b^	0.72^a^
UW551ΔR23	1^a^	1^a^
UW551ΔR9	0.76^a^	1^a^
UW551ΔR15	0.9^a^	1^a^
UW551ΔR27	0.83^a^	0.93^a^

^a,b^ denotes the statistical group that the strains belong. Statistics were calculated using data from multiple experiments of controls plus experimental strains, using one-way ANOVA analysis and Tukey HSD test. P < 0.01. For UW551ΔR13, five separate experiments totaling 48 plants for cool and 30 plants for warm temperatures were compared. UW551ΔR9 and UW551ΔR27 had three separate experiments totaling 29 plants for cool and 15 plants for warm. UW551ΔR23 had one experiment totaling 7 plants for cool and 6 plants for warm. UW551ΔR15 had one experiment totaling 10 plants for cool and 5 for warm. All experiments had equal number of plant controls inoculated with the wt *R*. *solanacearum* strains UW551 and K60, respectively, for statistical testing.

**Fig 2:**
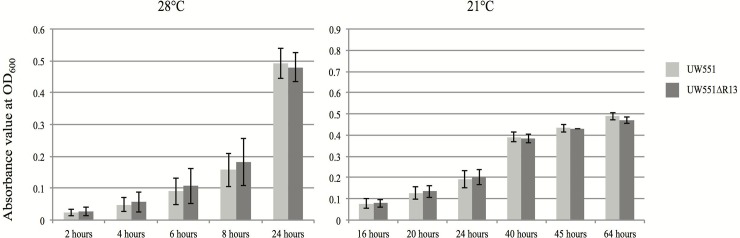
Comparison of in vitro growth between the wt strain UW551 and mutant strain UW551ΔR13 of *R*. *solanacearum* under warm (28°C, left) and cool (21°C) temperatures. Error bars indicate standard error. Significance of different OD_600_ values (P < 0.05) was measured using Student’s T-test.

To determine why the cool virulence of mutant strain UW551ΔR13 was different from the wt strain UW551, we measured and compared the growth rate, swimming, swarming, and twitching motilities of the two strains. In vitro growth at both cool (21°C) and warm (28°C) temperature conditions showed that the two strains had a similar growth rate at both temperatures (Fig 2).

Swimming and swarming motilities were also measured at 21°C and 28°C, and no significant difference was observed between the two strains at either temperature (Table 5).

**Table 5:** The wild type and the region 13 mutant of *R*. *solanacearum* had similar swimming and swarming motility at 21°C and 28°C.

Strain	Motility*			
	Swimming		Swarming	
	21°C	28°C	21°C	28°C
UW551	4.04±1.04	6.94±0.78	2.36±0.65	4.13±1.08
UW551ΔR13	4.04±1.16	6.35±0.9	2.53±1.04	3.80±1.09

*Values are the means of colony diameter, measured in centimeters, of three separate experiments with three replicates each. Student’s T-test was used to measure statistical significance (*P* < 0.05).

**Fig 3:**
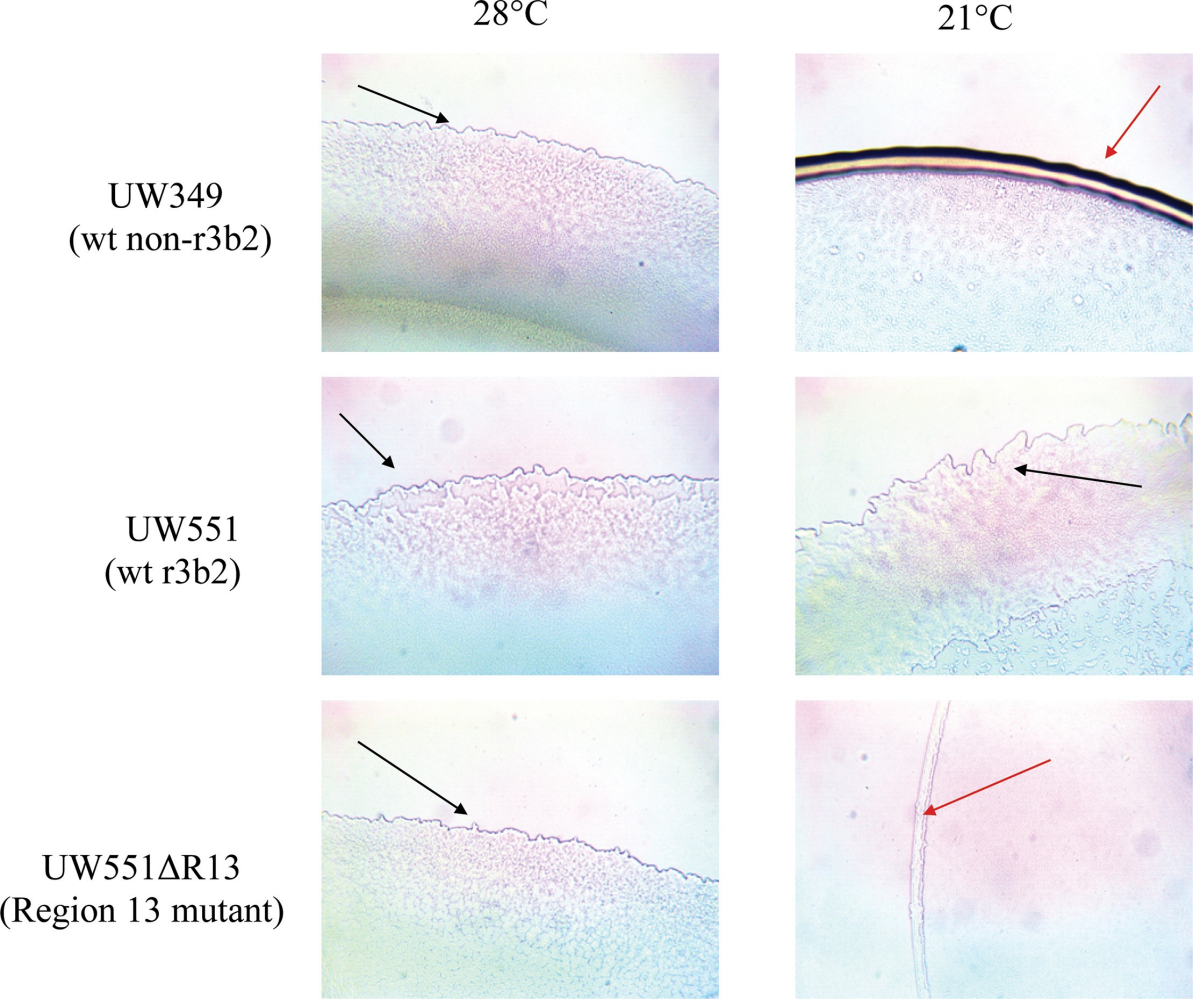
Twitching motility of *R*. *solanacearum* strains at 28°C and 21°C. The wt cool virulent r3b2 strain UW551 shows twitching at both temperatures (black arrows). The mutant strain UW551ΔR13, however, shows normal twitching at 28° (black arrow), but little twitching at 21°C (red arrow), similar to the wt non-cool virulent non-r3b2 strain UW349 (23-10BR).

Twitching motility was observed by monitoring the formation of corrugated trajectories or halos around colonies as the bacterium migrates over minimal media. The cool virulent r3b2 strain UW551 produced typical haloes under both cool and warm temperatures, while the non-cool virulent non-r3b2 strain UW349 (23-10BR) produced similar haloes at warm, but no haloes under cool temperature conditions (Fig 3). Mutant UW551ΔR13 displayed typical haloes as the wt strain at 28°C, but produced few halos at 21°C (Fig 3).

**Fig 4:**
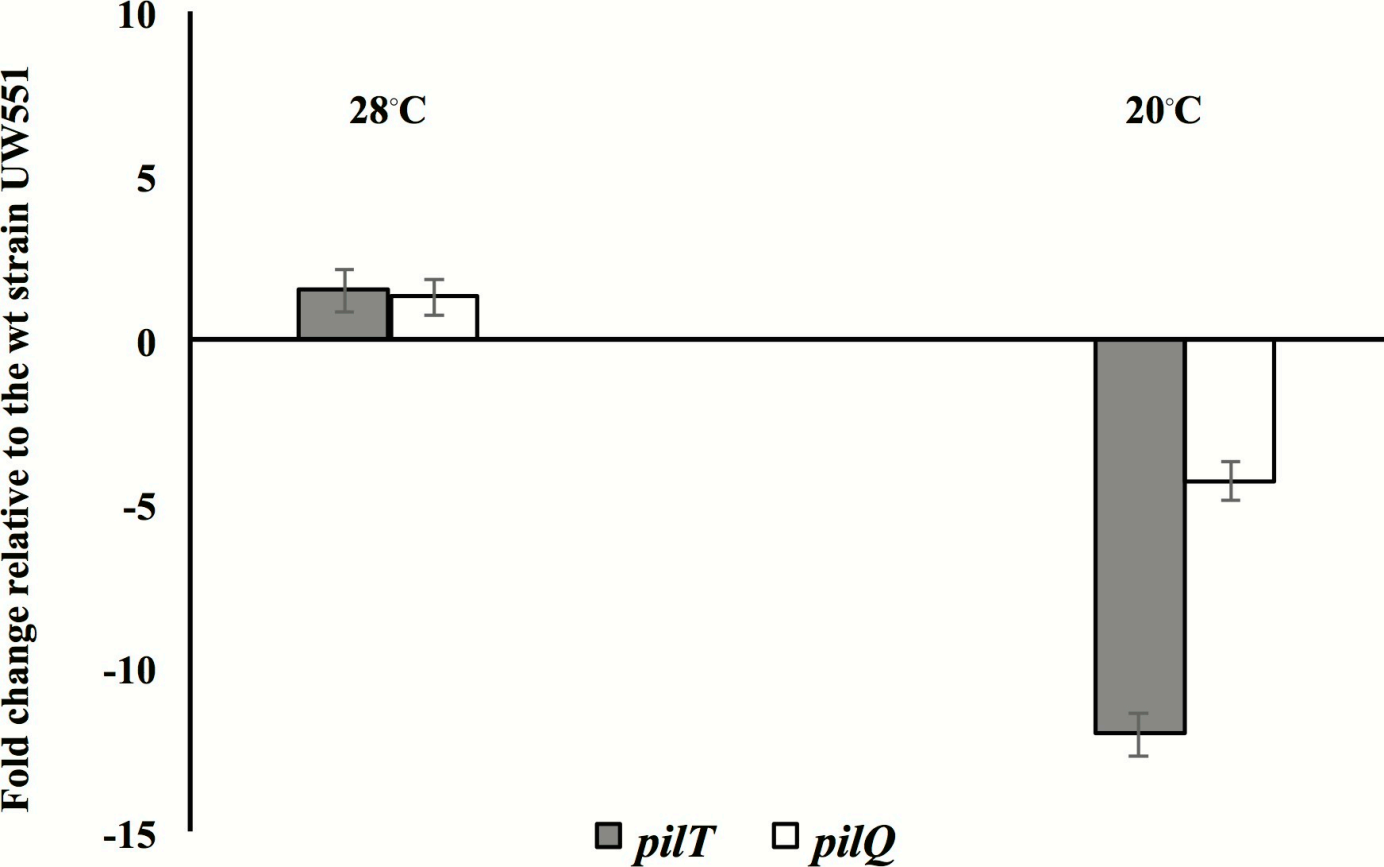
Fold-expression level changes of two genes, *pilT* and *pilQ*, in the mutant UW551ΔR13 relative to wt expression levels. Mutant UW551Δ13 had a 12.04-fold decrease in expression of the *pilT* gene relative to wt at 20°C, and a 4.34-fold decrease of *pilQ* at 20°C. These decreases were not observed at the warm temperature of 28°C.

The significantly reduced twitching motility of UW551ΔR13 was further tested by a root attachment assay and a comparative transcription assay of two genes, *pilT* and *pilQ*, involved in twitching motility. A similar percentage of the UW551ΔR13 cells attached to the roots of tomato seedlings at 28°C, but a significantly lower percentage of the cells attached to the roots at 20°C, as compared to the wt cells (Table 6). At the cooler temperature (20°C), *pilT* and *pilQ* expression levels in UW551ΔR13 were reduced by 12.04- and 4.34-fold, respectively, as compared to the wt expression levels (Fig 4). Under the warm temperature (28°C), expression levels in the mutant were partially increased by 1.5- and 1.3-fold, respectively (Fig 4).

**Table 6:** Percent of bacterial cells attached to the roots of tomato seedlings inoculated with the wt strain UW551 and the mutant strain UW551ΔR13 at 20°C and 28°C.

Strain	28°C^a^	20°C^a^
UW551	11.57% ±0.09	9.42% ±0.33
UW551ΔR13	11.34% ±0.51	3.24% ±0.31*

^a^Values were average percent of cells attached to the roots of tomato seedlings based on three separate experiments with 10 seedlings pooled in each experiment for each strain under each temperature.

* indicates P < 0.01 by the Student’s T-test.

Since region 13 significantly reduced virulence, twitching motility, and root attachment at cool temperatures, we designed the primer pair Rs-CV-F/Rs-CV-R based on the r3b2-unique sequence in this region, corresponding to nucleotides 73290 to 73309, and 73401 to 73422 in contig 0581 of UW551. The primer pair Rs-CV-F/Rs-CV-R amplified a 133-bp PCR band in all 34 r3b2 strains but in none of the 56 non-r3b2 strains of the *R*. *solanacearum* species complex, nor in any other non-*R*. *solanacearum* strains (Table 2). Unexpectedly, BLAST search at NCBI predicted that our Rs-CV primer set and previously published r3b2 primer sets—630/631 [21], B2 [22], RsSA [5], RsSA2 [11], RsSA3 [23] and RRSL2403 [24]—recognized two recently deposited Brazilian *R*. *solanacearum* strains RS488 (listed as a biovar 2T strain) and RS489 (listed as a biovar 1 strain) [18]. When the endoglucanase sequences of the two strains were analyzed using our *R*. *solanacearum* typing computer program [19], strain RS488 was placed into sequevar 1 clade, which does not indicate a change in specificity of all of the primer sets. Strain 489, however, is an anomaly. The phylogenetic tree constructed based on endoglucase sequences of *R*. *solanacearum* strains cannot place this strain into a known sequevar, yet it suggests this strain would be most likely related to strains in phylotype IIA (supposedly very distant than the r3b2 strains).

## Discussion

The goal of this study was to investigate whether any previously identified r3b2-unique fragments [5] are associated with cool virulence of r3b2 strains, and to use such a region for specific detection of this regulated group of *R*. *solanacearum*. We previously screened the UW551 genome for 500-bp unique sequences and identified 115 such fragments, and most of the fragments were adjacent to or contained hypothetical genes. When mapped back to the genome, some of these fragments form long, continuous regions that can be multiple kilobases in length, while others cluster into short, interspersed unique fragments along a multiple kilobase section of the genome. We identified 32 such regions in r3b2 genomes of *R*. *solanacearum*. As a result, grouped r3b2-unique regions, instead of individual fragments, can be deleted to save time and effort.

We generated five mutants of *R*. *solanacearum* by replacing 1,178 to 18,594 bp of the five selected regions with a 616-bp gentamicin cassette using site-directed marker-exchange mutagenesis. Four of the five mutants had no effect on any of the measured characteristics including biovar, virulence, growth rate, swimming and swarming motilities, and twitching motility at two temperature conditions.

We tested the mutants at two temperature conditions: “warm” ranging from 24 to 30°C in the greenhouse and 28 to 30°C in the laboratory to mimic the tropical lowland condition, and “cool” from 18 to 24°C in the greenhouse and 20 to 21°C in the laboratory to simulate the cool, temperate condition. One of the mutants, UW551ΔR13, missing region 13, showed biological variations, and the observed effects were cool-temperature dependent.

The region 13 deletion removed a genetic element that is in some way responsible for twitching motility specifically at cool temperatures. The swimming, swarming, and growth rate of this mutant remained unchanged, but twitching motility, root attachment, and twitching-related genes *pilT* and *pilQ* were all significantly reduced at the cooler temperature compared to the wt strain. This was also observed in the virulence assay, where a significantly reduced virulence was observed under cooler conditions, indicating this mutant behaved more similarly to non-r3b2 strains. Reduced twitching motility has been observed to cause a decrease in virulence previously, but those studies focused on the type IV pilus system and was not temperature dependent [25,26]. A proteomic comparison study of cool virulent and non-cool virulent strains, focusing on initial root colonization of tomato plants, suggested that the type VI secretion system may play a role in cool virulence [3]. Region 13 is located adjacent to a putative type VI VgrG-related tip protein and close to a putative RNA helicase gene. How the function(s) of this region is temperature dependent, however, is not easily explained, and needs further research.

A complementation of R13 was attempted but was unsuccessful, suggesting the region may need to be present in *cis* or is involved in *cis* regulation at cool temperatures. Future studies are needed to determine if any of the two hypothetical proteins in region 13 play a role in twitching motility and virulence at the cool temperature. In addition to region 13, future studies are also needed to determine whether any of the remaining 27 r3b2-unique regions are involved in cool virulence of r3b2 strains of *R*. *solanacearum*.

A PCR assay was developed by designing and validating primer pair Rs-CV-F/Rs-CV-R targeting region 13 for specific detection of r3b2 strains, since a loss of this region resulted in reduced cool virulence. The assay detected only the 34 r3b2 strains among the 95 tested *Ralstonia* and non-*Ralstonia* strains, confirming its specificity. This primer pair can be multiplexed easily with the *R*. *solanacearum* species- and plant-specific primer pairs RsSC-F/R and cox1-F/R [5] since these primers use similar PCR cycling parameters, enabling the detection of *R*. *solanacearum* at the species complex level, specifically identifying whether the strain is a cool virulent race 3 biovar 2 strain, and also excluding false negatives in a single reaction. It is unclear why Rs-CV and previously published r3b2 (sequevars 1&2)-specific primers all recognize the Brazilian *R*. *solanacearum* strain RS 489 by in silico prediction. Given the amount of genome similarity between strain RS 489 and the r3b2 strains, it would be fascinating to see a genome comparison. Curiously, however, RS 489 is absent from the NCBI dendrogram based on genomic BLAST analysis. Further study is needed to receive and test the RS489 strain for cool virulence and assay specificity before making any definitive claims. Nevertheless, our proposed assay still benefits researchers and regulators since among all the r3b2-specific PCR assays developed so far [5,11,21,22,23,24], this assay is the first to target a region demonstrated to be associated with the cool virulence-related function of r3b2 strains, which will facilitate accurate detection and exclusion of the select agent pathogen from the United States or from other countries where r3b2 is a quarantined pathogen.

## Conclusions

We demonstrated the association of genomic region 13 with cool virulence of the r3b2 strain UW551 of *R*. *solanacearum*. We also developed a PCR assay targeting a 133-bp sequence in region 13 for specific detection of *R*. *solanacearum* r3b2 strains. Future research is needed to elucidate the pathway(s) region 13 influences for a better understanding of the cool virulence of *R*. *solanacearum* r3b2 strains as select agent or quarantined pathogens.

## Supporting information

S1 Table Primers used to generate mutant strains of *R*. *solanacearum*. (DOCX)

S1 Raw Data File (XLSX)
