# Supplementary material for: Identification of a DNA region associated with the cool virulence of Ralstonia solancearum strain UW551 and its utilization for specific detection of the bacterium’s race 3 biovar 2 strains
Source: PLoS One. 2018 Nov 14;13(11):e0207280. doi: 10.1371/journal.pone.0207280 (PMC6235357; doi:10.1371/journal.pone.0207280)
Supplement: S1 Table — (DOCX) [file pone.0207280.s001.docx]

S1 Table. Primers used to generate mutant strains of *R. solanacearum*.

| **Primers** | **Sequence 5'-3', restriction enzyme sites are underlined** | **Amplicon** | **Mutated region (UW551 contigs)** |
| --- | --- | --- | --- |
| R13_up_F | GCTCTAGAACAACTCGAACCATAAGCCG *Xba*I | 1111bp | contig0581 (69877-70987) |
| R13_up_R | ATAAGAATGCGGCCGCTTTCTTCGCTTCCTCTGAGC *Not*I |  |  |
| R13_down_F | GGACTAGTGGTGTTTCGAGTATAGGCCG *Spe*I | 1058bp | contig0581 (74395-75452) |
| R13_down_R | GGGGTACCCTCATCGAACACCAGCATGT *Kpn*I |  |  |
| R27_up_F | GCTCTAGACACGCTTCTTTTCGTCGATT *Xba*I | 962bp | contig0581 (133555-132594) |
| R27_up_R | CCGCTCGAGGCCTGACGACATTTGAGGAA *Xho*I |  |  |
| R27_down_F | GGACTAGTGCATTATCCCAGCAATCACC *Spe*I | 1118bp | contig0581 (136276-135159) |
| R27_down_R | CGGGATCCCGGGTCTCTTCAGATTTCCA *Bam*HI |  |  |
| R9_up_F | GCTCTAGAGCTTGGTTCTTCCACGGC *Xba*I | 1082bp | contig0561 (34615-35696) |
| R9_up_R | CCGCTCGAGCACAGCGGATTGACACACTC *Xho*I |  |  |
| R9_down_F | CGGGATCCGCTGTGGGTTTGATGAGGTT *Bam*HI | 1286bp | contig0581 (143512-144797) |
| R9_down_R | GGGGTACCTTCCGATACTCAGCCCAGTT *Kpn*I |  |  |
| R15_up_F | AAACTGGTACCACTTGTGCAGAGTGCTTGG *KpnI* | 993bp | contig0535 (236-1228) |
| R15_up_R | TTACTGGATCCATCCTGGGTGAGGCCCTTAG *BamHI* |  |  |
| R15_low_F | ACAATGCGGCCGCAATGCCATGAGCCGCCAAG *NotI* | 960bp | contig0535 (7371-8352) |
| R15_low_R | TATATTCTAGACGACTCCGATATGCGCCTTC *XbaI* |  |  |
| R23_up_F | GCTCTAGAGCGCTGATTTCGCTCTATCT *XbaI* | 888bp | contig0571 (79806-80693) |
| R23_up_R | CCGCTCGAGCAACTGGGTGCTATTTGGCT *XhoI* |  |  |
| R23_down_F | CGGGATCCGCCTTGAGCAAAGATTCTGG *BamHI* | 919bp | contig0571 (81872-82790) |
| R23_down_R | GGGGTACCGCAATACCTGGTGCTGTTCA *KpnI* |  |  |
